# Real-World Data on Apatinib Efficacy - Results of a Retrospective Study in Metastatic Breast Cancer Patients Pretreated With Multiline Treatment

**DOI:** 10.3389/fonc.2021.643654

**Published:** 2021-06-10

**Authors:** Zhaoyun Liu, Jing Shan, Qian Yu, Xinzhao Wang, Xiang Song, Fukai Wang, Chao Li, Zhiyong Yu, Jinming Yu

**Affiliations:** ^1^ Department of Radiation Oncology, Shandong Cancer Hospital and Institute, Cheeloo College of Medicine, Shandong University, Jinan, China; ^2^ Shandong Cancer Hospital and Institute, Shandong First Medical University and Shandong Academy of Medical Sciences, Jinan, China; ^3^ Department of Surgery, Cleveland Clinic, Cleveland, OH, United States

**Keywords:** breast cancer, apatinib, metastatic, efficacy, safety

## Abstract

**Objectives:**

The NCCN guidelines recommend that the addition of bevacizumab should be considered in metastatic breast cancers in some circumstances, but there are no recommendations for the similar antiangiogenic drug apatinib. The aim of this study was to evaluate the safety and efficacy of apatinib in metastatic breast cancer patients pretreated with multiline treatment in a real-world setting.

**Materials and Methods:**

Metastatic breast cancer patients pretreated with multiline treatment who had apatinib treatment initiated from September 2015 to August 2019 at Shandong Cancer Hospital and Institute were included. The primary endpoints included PFS and OS, and the secondary endpoint was treatment-related toxicity.

**Results:**

A total of 66 patients with metastatic breast cancer received apatinib treatment after failure of multiline chemotherapy in this study. The median PFS and OS of all 66 patients were 6.0 months and 10.0 months, respectively. The clinical beneficial rate was 40.9%. All patients tolerated treatment well, and no patients died of toxicity. The common toxicities of apatinib were hand and foot syndrome, secondary hypertension and fatigue events. The number of prior chemotherapy regimens was significantly associated with DFS and OS. Capecitabine may be a better choice for combination with a longer median OS of 19 months, while apatinib combined with other drugs was 9 months, and the apatinib monotherapy was 10 months.

**Conclusion:**

Apatinib produced moderate efficacy in metastatic breast cancer patients pretreated with multiline treatment with no significant treatment-related adverse events. Apatinib might be a choice for women as a maintenance salvage therapy following multiline chemotherapy failure.

## Introduction

The incidence of breast cancer in the world was 1.9 million in 2017 in women, with an annual mortality of 601000 accounting for the number of deaths in females. Four percent of newly diagnosed breast cancer patients present with metastatic disease (1), which is associated with a poor prognosis and a median overall survival (OS) less than 3 years despite treatment (2). For metastatic breast cancer patients pretreated with multiline treatment with drug resistance to chemotherapy, it is a considerable challenge to choose an appropriate treatment due to the lack of standard treatment (3). Angiogenesis is closely associated with tumor growth and metastasis (4). As the first anti-VEGF agent, bevacizumab was recommended in the 2019 NCCN guidelines in certain circumstances because of the improved PFS in randomized clinical trials with the addition of bevacizumab to some first- or second-line treatments for metastatic breast cancer (5). This evidence suggests that anti-angiogenic drugs may be effective in a certain subtype of breast cancer patients.

Apatinib is a novel VEGFR inhibitor that targets VEGFR-2, which contributes to angiogenesis. It was first recommended in advanced gastric or gastroesophageal junction adenocarcinoma patients in 2014 (6), and it has also been studied in liver cancer, cervical cancer, and lung cancer (7–9). A previous phase II clinical trial showed the efficacy and safety of apatinib monotherapy in metastatic heavily pretreated TNBC patients (10). There is only a small real-world study on the efficacy and safety of apatinib in 85 patients with advanced breast cancer (11). It is necessary to evaluate the effects of apatinib in breast cancer patients more accurately in a real-world setting.

We present this study to conduct a retrospective evaluation of the efficacy and toxicity of apatinib monotherapy or apatinib combined with other chemotherapeutic drugs after heavy pretreatment chemotherapy as a rescue in metastatic breast cancer in clinical practice.

## Materials and Methods

### Patients

In this study, we retrospectively collected clinicopathological information and follow-up records from 66 breast cancer patients who received apatinib treatment between September 2015 and August 2019 at the Shandong Cancer Hospital and Institute. Patients who had finished more than one month of apatinib treatment were included in this study. The last follow-up date was February 2020. This study obtained approval from the Ethics Committee of the Shandong Cancer Hospital and Institute and was approved to meet the standard of clinical practice.

The criteria for enrollment in the group were as follows: age ≥ 18 years; pathologically confirmed breast cancer with local or distant organ metastasis; apatinib regimen administered after failure of second-line rescue chemotherapy; and patients with follow-up records. All patients were given apatinib orally as a daily monotherapy or in combination with chemotherapeutic drugs. The drug dose varied according to the physical condition of the individual, and 850 mg, 500 mg, 425 mg, and 250 mg doses were used. The dose was eventually reduced to 250 mg for patients with intolerant side effects for high-dose apatinib. Assessments were evaluated every two or three rescue treatments. Disease-free survival (DFS) was defined as the time from the oral administration of apatinib to the first progression and was assessed per the RECIST criteria of the disease (12). The OS was considered the interval from the oral administration of apatinib until death.

### Statistical Analysis

Categorical and continuous variables are reported as frequencies and the mean/median with standard deviation, respectively. OS and PFS were plotted with Kaplan-Meier curves, which were compared with log-rank tests. A Cox proportional hazard ratio regression model was used to determine predictors of survival; statistically significant variables in the univariate analyses were then analyzed with a stepwise multivariate analysis. SPSS statistics version 17.0 or GraphPad Prism version 8.0 were used for all statistical analyses.

## Results

### Patient Characteristics

A total of 66 metastatic breast cancer patients treated with apatinib were included. The clinical beneficial rate (CBR) was 40.9% (27/66), with a median PFS of 6.0 months and a median OS of 10.0 months. The characteristics of clinical data and some hematological results at baseline are summarized in **Table 1**. The median age at first diagnosis of breast cancer was 45 years (ranging between 28 and 70 years). TNBC patients accounted for 48.5% (32/66). Approximately 66.7% (44/66) of patients were premenopausal women at first diagnosis. The ECOG status was 0–1 in 39 patients (59.1%) and 2 in 21 patients (31.8%). At the first diagnosis, 51.5% patients were diagnosed with stage I-II disease, 39.4% with stage III disease, and 9.1% with stage IV disease. The majority of primary recurrences occurred in the viscera or bone (83.3%). Before apatinib treatment, there was more than one organ metastasis (60.6%), including liver metastasis in 20 cases (30.3%), brain metastasis in 12 cases (18.2%), lung metastasis in 46 cases (69.7%), bone metastasis in 34 cases (51.5%), and lymph node metastasis in 39 cases (59.1%). The patients who received the lowest dose of 250 mg of apatinib accounted for 66.7%. Of the 22 patients who were treated with apatinib at a dose of 425 mg, 500 mg or 850 mg. 13 patients were eventually given a reduction to 250 mg because of side effects. Approximately 33.3% were treated with apatinib alone.

**Table 1:** Patient characteristics.

Characteristics	Number N = 66	Percentage (%)
ECOG performance status		
0-1	39	59.1
2	21	31.8
3	6	9.1
Stage disease of first diagnosis		
I-II	34	51.5
III	26	39.4
IV	6	9.1
Histology		
Invasive cancer	59	89.4
No Invasive cancer	7	10.6
Subtype		
TNBC	32	48.5
No-TNBC	34	51.5
Site of primary recurrence		
Local	11	16.7
Viscera or bone	55	83.3
Number of organs in primary recurrence		
≤1	42	63.6
>1	24	36.4
Number of organs before apatinib		
≤1	26	39.4
>1	40	60.6
First-line rescue effect		
PD	27	40.9
PR/SD	39	59.1
Number of prior chemotherapy regimens		
≤3	23	34.8
>3	43	65.2
Metastasis site before apatinib		
Liver	20	30.3
Brain	12	18.2
Lung	46	69.7
Bone	34	51.5
Nodes	39	59.1
other	6	9.1
Apatinib dose (mg)		
250/ reduce the amount to 250	44/13	66.7/19.7
425/500/850	22	33.3
Dose reduction		
Yes	13	19.7
No	53	80.3
Apatinib monotherapy		
Yes	22	33.3
No	44	66.7
United capecitabine		
Yes	14	21.2
Other	30	45.5
alone	22	33.3
Age, years	45.61±10.88	
Ki67	40.52±26.65	
Month interval between initial recurrence	35.55±40.56	
NLR	3.77±3.48	
PLR	231.32±156.52	
WBC, ×10^9^	5.63±2.65	
Mononuclear cell, ×10^9^	0.52±0.34	
RBC, ×10^12^	5.13±8.99	
Hemoglobin	120.26±14.43	

### Treatment-Related Adverse Events

Most patients were treated with the lowest dose of 250 mg of apatinib (66.7%). Among patients treated with high doses, 59.1% patients (13/22) were treated with a reduction to 250 mg of apatinib. All patients tolerated treatment well without toxicity-related death. Grade 1–2 toxicities were the common; the rate of grade 3 toxicity was 10.6% (7/66). Grade 4 toxicity was not observed. Hand and foot syndrome (25.8%), secondary hypertension (22.7%), fatigue (16.7%), and pain (13.6%) events were the common toxicities (**Table 2**).

**Table 2:** Treatment-Related Toxicities.

Adverse Event	Grade1-2	Grade3
Hand and foot syndrome	17 (25.8%)	4 (6.1%)
Secondary hypertension	15 (22.7%)	2 (3.0%)
Fatigue	11 (16.7%)	1 (1.5%)
Pain	9 (13.6%)	0
Oral mucositis	5 (7.6%)	0
Diarrhea	3 (4.5%)	0
Liver dysfunction	2 (3.0%)	0
Nausea	2(3.0%)	0
Hemorrhage	2(3.0%)	0

### Factors Correlated With PFS and OS

**Figure 1:**
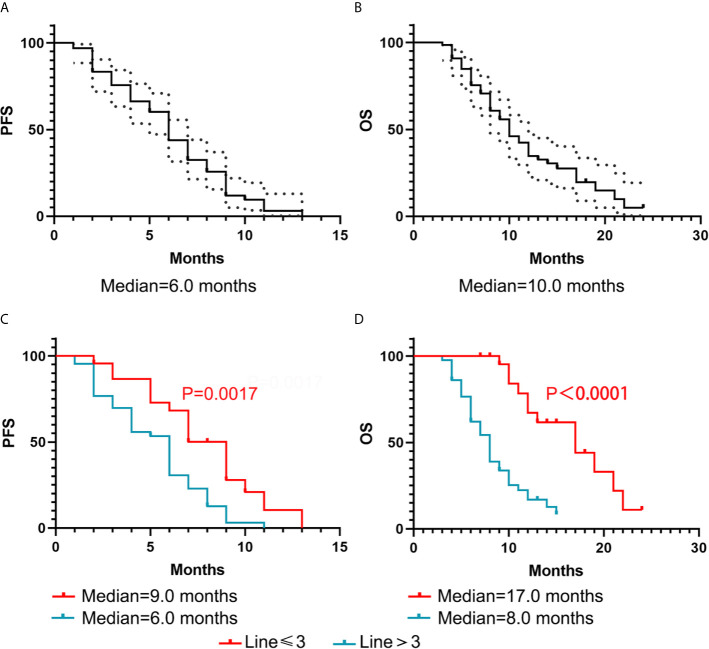
Kaplan–Meier curve of PFS and OS for all 66 patients. **(A, B)** Black-dotted lines represent 95% confidence intervals (CIs). **(C, D)** The association with the number of prior chemotherapy regimens and DFS and OS.

The median PFS of all 66 patients was 6.0 months (95%CI, 5.249-6.751). The median OS of all 66 patients was 10.0 months (95%CI, 8.104-11.896) (**Figure 1**). As shown in **Table 3**, in the multivariate analysis, the number of prior chemotherapy regimens was significantly associated with DFS (P = 0.028) and OS (P = 0.002). Brain metastasis (P = 0.045) was significant in the univariate analysis but did not retain significance in the multivariate analysis of PFS. The neutrophil-to-lymphocyte ratio (NLR) (P = 0.0001), subtype (P = 0.048), number of organs involved in the primary recurrence (P = 0.033), and number of organs involved before apatinib treatment (P = 0.048) were significant in the univariate analysis but not in the multivariate analysis of OS.

**Table 3:** Analysis of correlation factors with PFS and OS.

Characteristics	PFS						OS					
	Univariate analysis			Multivariate analysis			Univariate analysis			Multivariate analysis		
	HR	95% CI	P Value	HR	95% CI	P Value	HR	95% CI	P Value	HR	95% CI	P Value
NLR	1.033	0.944 to 1.131	0.481				1.160	1.073 to 1.253	0.0001	1.057	0.887 to 1.259	0.534
PLR	1.001	0.999 to 1.003	0.229				1.001	1.000 to 1.003	0.119			
NAC	1.187	0.611 to 2.307	0.614				0.955	0.422 to 2.158	0.911			
Histology	1.745	0.629 to 4.841	0.284				1.119	0.399 to 3.136	0.831			
Stage disease of first diagnosis	0.837	0.517 to 1.356	0.469				1.206	0.748 to 1.944	0.443			
Ki67	1.008	0.997 to 1.019	0.160				0.997	0.986 to 1.007	0.550			
Subtype	1.365	0.798 to 2.335	0.256				1.859	1.004 to 3.440	0.048	1.916	0.889 to 4.132	0.097
Interval between initial recurrence	0.996	0.989 to 1.003	0.273				0.999	0.992 to 1.006	0.770			
Site of primary recurrence	1.208	0.590 to 2.475	0.605				0.726	0.348 to 1.513	0.392			
Number of organs in primary recurrence	0.823	0.479 to 1.415	0.481				0.521	0.287 to 0.948	0.033	0.962	0.389 to 2.379	0.933
Number of organs before apatinib	0.862	0.501 to 1.482	0.591				0.547	0.301 to 0.995	0.048	0.658	0.229 to 1.892	0.437
First-line rescue effect	1.103	0.653 to 1.864	0.714				1.032	0.559 to 1.904	0.919			
Number of prior chemotherapy regimens	2.265	1.262 to 4.064	0.006	1.971	1.077 to 3.607	0.028	5.525	2.425 to 12.585	0.0001	4.555	1.718 to 12.080	0.002
Brain Metastasis	2.090	1.016 to 4.299	0.045	1.731	0.833 to 3.596	0.142	1.793	0.879 to 3.657	0.109			
Lung Metastasis	1.151	0.660 to 2.008	0.620				0.812	0.443 to 1.490	0.501			
Liver Metastasis	0.750	0.422 to 1.334	0.328				1.431	0.780 to 2.625	0.247			
Apatinib dose	1.012	0.581 to 1.764	0.966				0.607	0.306 to 1.202	0.152			
Apatinib monotherapy	0.725	0.422 to 1.243	0.242				0.721	0.407 to 1.279	0.264			
United capecitabine	0.922	0.666 to 1.276	0.625				0.964	0.676 to 1.375	0.841			

**Figure 2:**
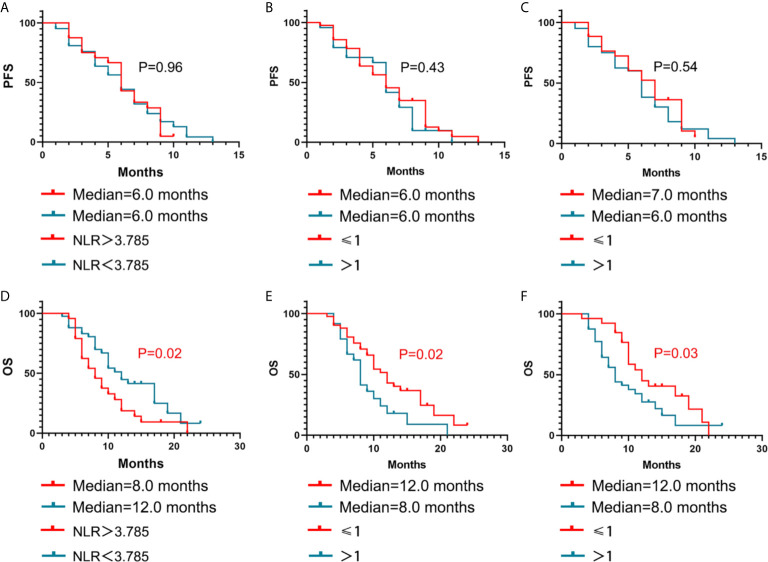
Kaplan–Meier estimates of PFS and OS with different influencing factors. NLR **(A, D)**, number of organs involved in the primary recurrence **(B, E)**, and number of organs involved before apatinib treatment **(C, F)**.

**Figure 3:**
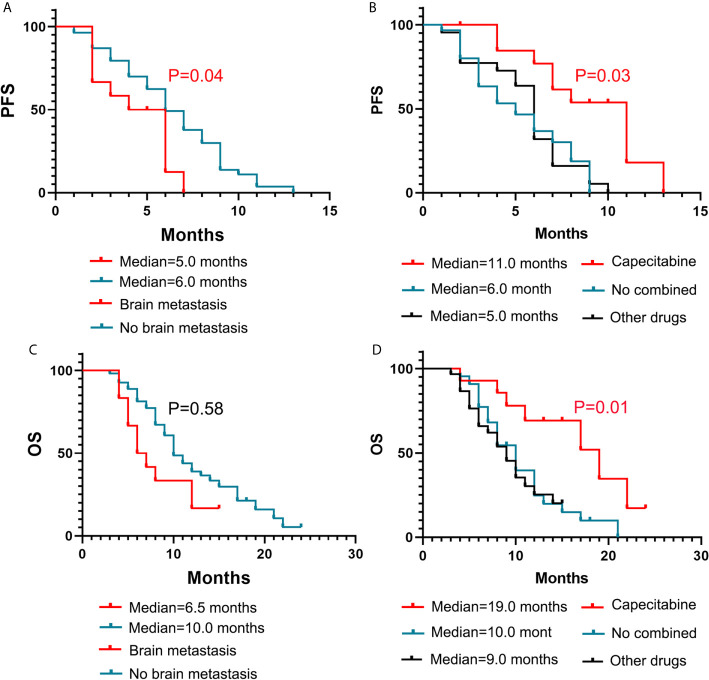
Kaplan–Meier estimates of PFS and OS with different influencing factors. Brain metastasis **(A, C)** and different drug regimens **(B, D)**.

The Kaplan-Meier curves for PFS and OS based on the NLR, number of organs involved in the primary recurrence, number of organs involved before apatinib treatment, are displayed in **Figure 2**. The Kaplan-Meier curves for PFS and OS based on the brain metastasis and the different combined methods are displayed in **Figure 3**. In **Table 4**, a number of prior chemotherapy regimens less than or equal to 3 seemed to be associated with a longer PFS (9 *vs*. 6 months, P = 0.0017) or OS (17 vs. 8 months, P < 0.0001). Patients with brain metastasis had a poorer prognosis in terms of DFS (5 *vs*. 6 months, P = 0.0472) and OS (6.5 vs. 10.0 months, P=0.0303). The patients with an NLR>3.78 (P=0.0221), a number of organs in primary recurrence>1 (P = 0.0238), a number of organs >1 before apatinib (P = 0.0365), had a poorer OS (P < 0.05). Apatinib were shown to have more benefit when used in combination with capecitabine compared with apatinib monotherapy or combined with other drugs. When apatinib combined with capecitabine, the median OS was 19 months, while apatinib combined with other drugs was 9 months, and the apatinib monotherapy was 10 months.

**Table 4:** Information about possible factors related to PFS and OS.

		PFS		OS	
		mPFS (month)	P	mOS (month)	P
NLR	<3.78(42)	6	0.9637	12	0.0221
	>3.78(24)	6		8	
Number of organs in primary recurrence	≤1(41)	6	0.4324	12	0.0238
	>1(25)	6		8	
Number of organs before apatinib	≤1(26)	7	0.5479	12	0.0365
	>1(40)	6		8	
Number of prior chemotherapy regimens	≤3 (23)	9	0.0017	17	<0.0001
	>3(43)	6		8	
brain metastasis	Yes(12)	5	0.0472	6.5	0.0303
	No(54)	6		10	
different drug regimens	Capecitabine (14)	11	0.5876	19	0.0111
	Other drugs (30)	5		9	
	No combined (22)	6		10	
Whether achieved remission	Yes (27)	9	<0.0001	12	P=0.0017
	No (39)	4		8	

The patients who achieved clinical benefits had a better prognosis with the median OS and PFS were 12 months and 9 months while the patients with no clinical benefits was 8 months and 4 months (all P < 0.05, **Table 4**).

## Discussion

Metastatic breast cancer is an incurable disease with a median survival time of 2-3 years, and failure after more than second-line therapy chemotherapy often means a poor prognosis, with a median PFS of less than 4.2 months with traditional chemotherapy (13, 14). The metastatic breast cancer patients treated with single chemotherapy that had not been used before had shown PFS of 2-5 months (15). The development and progressive growth of the tumor is closely related to the aberrant process of angiogenesis (16). It may be an important treatment approach to inhibit tumor angiogenesis (17). VEGFR2 is involved in pro-angiogenic signaling and promotes the formation of lymphatic vessels and blood vessels (18). Apatinib, an oral tyrosine kinase inhibitor targeting VEGFR2, plays a vital role in tumor angiogenesis. It was reported that apatinib inhibits proliferation and migration *via* an anti-angiogenic mechanism not by direct inhibition of the tumor cells (19). Preclinical data have shown that apatinib inhibited the growth of xenograft tumors either alone or in combination with chemotherapeutic drugs (20). Clinically, apatinib monotherapy or apatinib in combination with chemotherapy is an alternative therapy.

Apatinib was first approved to be used in the treatment of advanced or metastatic gastric cancer by the Chinese Food and Drug Administration in 2014 with the prolonged median PFS and OS compared with placebo in prospective phase 2 and 3 trials and real-world studies (21, 22). There are also limited clinical trials in other advanced or metastatic cancers, such as lung cancer, liver cancer, colorectal cancer, osteogenic sarcoma, and breast cancer (9, 23–25). A prospective study reported that apatinib had encouraging clinical activity with a median PFS of 5.4 months and a median OS of 10.0 months in extensive-stage small-cell lung cancer patients who had more than two prior chemotherapy treatment failures with manageable toxicity (26). The combination of an anti-PD-1 antibody (SHR-1210) and apatinib (250 mg) treatment showed promising efficacy with acceptable toxicity in patients with hepatocellular carcinoma, gastric cancer, esophagogastric junction cancer, and advanced non-squamous NSCLC by modulating tumor immune microenvironment (27, 28). A retrospective study of 23 metastatic colorectal cancer patients demonstrated that the combination of apatinib with chemotherapy was more effective treatment than apatinib monotherapy (29, 30).

Our results were nearly consistent with the clinical trials and current observational study with the median PFS and OS were 6.0 months and 10.0 months, respectively. Two phase II clinical trials on the use of apatinib in metastatic breast cancer were published in 2014 (10, 31). The dose of 500 mg of apatinib was safe, and the partial response rate was encouraging in metastatic TNBC and non-triple-negative breast cancer. In this prospective clinical trial, the median PFS and median OS were 3.3 months and 10.6 months, respectively, and the CBR was 25.0% in heavily pretreated metastatic TNBC patients (10). In the current observational study, metastatic breast cancer patients who were considered intolerant for standard treatments before apatinib treatment achieved a median PFS of 4.9 months and OS of 10.3 months (32). In our retrospective study, we found that the CBR was 40.9%, with a median PFS of 6.0 months and a median OS of 10.0 months in all 66 patients. The effect on the CBR and PFS were slightly better than those in phase II clinical trials and the observational, which may be due to the difference in treatment regimens. In this study, most patients received apatinib combined with chemotherapy, while phase II clinical trials and the observational only used apatinib alone, indicating that the combination of chemotherapy and apatinib is better than apatinib monotherapy, despite the fact that direct numerical comparison may not be appropriate.

It may provide better effect with the combination of apatinib and conventional chemotherapy for advanced breast cancer patients following multiline chemotherapy failure (33). According to the results of a preclinical study, apatinib reduces neovascularization while inducing normalization of tumor blood vessels and facilitating the delivery of cytotoxic drugs (20). We conclude that apatinib combined with chemotherapy drugs might offer a more effective treatment option than single-agent therapy, and apatinib combined with capecitabine was first recommended in metastatic patients. However, it is possible that most of the patients treated in combination with capecitabine had not been treated with capecitabine before when they received the rescue chemotherapy, and the number of lines of treatment with apatinib was less than that of capecitabine combined with other drugs. More clinical trials are needed for further verification.

In this real-world retrospective study, the differences in chemotherapy drugs may increase the occurrence of toxicity. However, all patients were tolerated treatment well, and no patients died of AEs. The most frequent toxicities of apatinib in this study were hand and foot syndrome, secondary hypertension, fatigue, and pain events. Most patients were treated with the lowest dose of 250 mg of apatinib in this study, and of the 22 patients who were treated with apatinib at a dose of 425 mg, 500 mg or 850 mg, 13 patients were eventually given a reduction to 250 mg because of side effects. The lower dose of apatinib used in this retrospective research was effective, and the prognosis was not worse than that in some high-dose studies that used 500 mg/day (10, 31). Although it has been reported that the toxicity of 500 mg apatinib is tolerable, our research found that for patients who have received multiline rescue chemotherapy at a later stage, many patients may require a low dose of 250 mg for maintenance because of poor individual status.

We found the nearly efficacy and safety of the apatinib therapy compared with phase II trials. However, this is a real-world study which are more complicated than prospective study. The patients’ status in our study was much worse than phase II trials. 65.2% of patients received>3 lines of apatinib treatment while the corresponding rate in phase III trial was 7.9% (31). We found that the number of prior chemotherapy regimens significantly associated with DFS and OS. The PFS was 9.0 months and 6.0 months in patients with ≤ 3 and >3 lines before apatinib, respectively. The OS was 17.0 months and 8.0 months in those with ≤ 3 lines and >3 lines before apatinib, respectively. Current treatments in metastatic breast cancer patients are still inadequate, and patients who have failed with less than third-line treatments require additional choices that are usually made based on each physician’s experience and each patient’s status. We hypothesize that apatinib may be circumvent multidrug resistance in patients with conventional chemotherapy drugs, and the earlier apatinib is applied, the better the prognosis in advanced breast cancer.

Overall, patients with metastatic breast cancer who were pretreated with multiline treatment may benefit from apatinib in terms of PFS and OS. All patients tolerated treatment well, and no patients died of AEs. However, as a retrospective study, there were some limitations in our study. Further clinical practice and long follow-up data are required. We are recruiting patients for a prospective analysis to validate its efficacy in the treatment of metastatic breast cancer.

## Conclusions

In this study, we analyzed patients with metastatic breast cancer who had experienced apatinib treatment after failed multiline therapy. It demonstrated that apatinib may be an effective treatment for patients with metastatic breast.

## Data Availability Statement

The raw data supporting the conclusions of this article will be made available by the authors, without undue reservation.

## Ethics Statement

The studies involving human participants were reviewed and approved by the Ethics Committee of the Shandong Cancer Hospital and Institute. The patients/participants provided their written informed consent to participate in this study.

## Author Contributions

ZL analyzed data and wrote the manuscript. JS collected the data. QY and XW analyzed data. XS, CL, and FW edited the manuscript. ZY and JY played a role in developing the idea. All authors agree with the order of presentation of the authors. All authors contributed to the article and approved the submitted version.

## Funding

This work was funded by the grants from National Key Research and Development Projects of China (2018YFC1312201), the grants from the Natural Science Foundation of Shandong Province (ZR2019MH109, ZR2017PH055).

## Conflict of Interest

The authors declare that the research was conducted in the absence of any commercial or financial relationships that could be construed as a potential conflict of interest.
